# G-Protein-Coupled Receptor Kinase 2 Limits CCL21-Induced T Cell Migration via Phospholipase Cγ1

**DOI:** 10.3390/receptors4030017

**Published:** 2025-09-03

**Authors:** Anahi Sanchez, Caitlin T. Winebrenner, Natalia Garcia, Brian Kaiser, Lyndsey Kilgore, Cesar I. Cardona, Daniel W. Bassuk, Mary E. Miller, Charles A. Bill, Laura A. Shannon, Brant M. Wagener, Amy Wagler, Manuel Llano, Colin A. Bill, Charlotte M. Vines

**Affiliations:** 1 Department of Biological Sciences, The University of Texas at El Paso, El Paso, TX 79968, USA;; 2 Department of Microbiology, Genetics and Immunology, The University of Kansas Medical Center, Kansas City, KS 66103, USA;; 3 Department of Anesthesiology and Perioperative Medicine, Heersink School of Medicine, University of Alabama Birmingham, Birmingham, AL 35294, USA;

**Keywords:** G protein-coupled receptor kinase, ligand-biased signaling, chemokine, T cell

## Abstract

**Background/Objectives:**

G protein-coupled receptors (GPCRs) can promote ligand-biased signaling, yet the mechanisms that promote bias are not well understood. We have shown that C-C Chemokine Ligand 19 (CCL19) and CCL21 promote ligand-biased internalization and signaling of C-C Chemokine Receptor 7 (CCR7) in T cells. The roles of GPCR kinases (GRKs) in regulating biased CCR7 internalization and biased signaling in T cells are unclear. GRK2 is a serine/threonine kinase that phosphorylates GPCRs in response to ligand binding and is recruited to the plasma membrane via its C-terminal pleckstrin homology domain to phosphatidylinositol 4,5-bisphosphate (PIP_2_).

**Methods:**

Human embryonic kidney cells (HEK293) transfected to express wild-type and mutant GRK2 and human CCR7, human T cell lines harboring heterozygous deletions of GRK2, and naïve primary T cells from GRK2 heterozygous (GRK2^+/−^) or GRK2^f/f^ CD4-Cre mice were used to examine the effects of GRK2 on ligand-induced CCR7 signaling in T cells. We used flow cytometry to assay the effect of GRK2 on CCR7 internalization, Fluorescence Resonance Energy Transfer (FRET) to define the effect of GRK2 on CCR7 activation of Gα_i_ isoforms and transwell migration assays to examine the effect of GRK2 on chemotaxis. Since chemotaxis via CCR7 is mediated by phospholipase Cγ1 (PLCγ1), Western blot assays were used to measure the effect of GRK2 during downstream signaling via phosphorylation of PLCγ1.

**Results:**

We found that following CCL19 binding, GRK2 promoted kinase-dependent CCR7 recruitment of arrestin-3, rapid CCR7 internalization and Gα_i3_ recruitment to CCR7. In contrast, following binding of CCL21 to CCR7, GRK2 slowed CCR7 internalization, induced recruitment of Gα_i2_ to the activated receptor_,_ and promoted chemotaxis. Since we have shown that CCL21 promotes chemotaxis via PLCγ1, we examined the effect of GRK2 on PLCγ1 activation and found that GRK2 had no effect on CCL21-mediated PLCγ1 phosphorylation.

**Conclusions:**

GRK2 promotes differential signaling downstream of CCR7 activation by CCL19 and CCL21 and provides a model for biased signaling downstream of a GPCR driven by GRK2.

## 1. Introduction

Signaling via G protein-coupled receptors (GPCRs), the largest group of membrane receptors in humans [1,2], is initially regulated by the rate of heterotrimeric G protein activation and the extent of GPCR phosphorylation [1,2]. Although GPCRs can undergo heterologous phosphorylation and become desensitized by different protein kinases, GPCR kinases (GRKs) are serine/threonine kinases that are the foremost enzymes promoting homologous GPCR phosphorylation and desensitization [3,4]. The seven known GRKs have been grouped into three classes based on their structures: (1) rhodopsin kinases (GRK1 and GRK7), (2) cytosolic β-adrenergic receptor kinases (β-ARKs (GRK2 and GRK3)), and (3) the membrane-tethered GRK4 family (GRK4, GRK5, and GRK6). While rhodopsin kinases regulate vision and GRK4 is expressed in the testes, uterus, brain, and kidney [5,6], GRKs 2, 3, 5, and 6 are ubiquitously expressed (reviewed in [7]). GRK-induced GPCR phosphorylation promotes β-arrestin recruitment to the cytoplasmic face of the GPCR, typically limiting further G protein activation [8]. However, in some instances, this activation step is independent of G proteins [9–15]. To selectively regulate GPCRs, it is important to identify signaling mechanisms that are controlled by GRK activity.

Arrestins can facilitate GPCR internalization via endosomes, where GPCRs are either degraded or recycled back to the membrane (reviewed by [16]). There are four arrestins, and two of these, arrestin-2 (β-arrestin-1) and arrestin-3 (β-arrestin-2), are ubiquitously expressed. Following ligand binding, GPCRs activate the coupled heterotrimeric G proteins, and GRKs phosphorylate the third intracellular loop and C-terminus, which can promote arrestin binding. The phosphorylation pattern, which determines arrestin conformation following GPCR binding, influences GPCR internalization, degradation, and recycling [17].

C-C chemokine receptor seven (CCR7) is a GPCR expressed by naïve and central memory T cells, and some activated immune cells [16,18–20]. CCR7 has two ligands, C-C chemokine ligand 19 (CCL19) and CCL21, that are expressed on thymic and lymphatic endothelia. Additionally, CCL19 is expressed by mature antigen-presenting cells [21,22]. Both CCL19 and CCL21 promote chemotaxis of CCR7-expressing immune cells to and through primary and/or secondary lymphoid tissues [18,19,23–25]. CCL19 and CCL21 share 32% identity, with the primary structural difference being an extra 37 amino acids in the extended, unstructured C-terminus of CCL21 that tethers the chemokine to cell surfaces and inhibits CCL21 activity [26–29]. CCL19 and CCL21 bind CCR7 with similar affinities (Kd ≈ 100 pM) and with equivalent efficacy and potency for the activation of the Gα_i/o_ subfamily of G proteins [30]. Interestingly, CCL19 and CCL21 promote distinct signaling events, which is a well-known phenomenon termed biased signaling [3,31–33]. We have shown that in primary naïve T cells, CCR7 binding to CCL19 promotes recruitment of arrestin-3, leading to strong downstream signaling via mitogen-activated protein kinases (MAPK), during internalization of ~80% of the CCR7 on the cell surface. In contrast, when CCR7 is bound to CCL21, only 20% of CCR7 on the cell surface is internalized, which shuts off MAPK signaling, and this internalization is β-arrestin independent [31,34].

Studies attempting to define a role for individual GRKs involved in CCR7 internalization and signaling have used non-lymphoid Human Embryonic Kidney (HEK293T) cells and found that exogenously expressed CCR7 is phosphorylated by GRK3 and GRK6 [30]. Using murine T cells, where CCR7 is expressed endogenously, GRK2 had minimal effect on CCL19-, or CCL21-induced chemotaxis but led to upregulation of the sphingosine-1 phosphate receptor [34,35]. Since the expression levels of GRK2 protein in immune cells can vary during different disease states, including cardiac failure, rheumatoid arthritis, and multiple sclerosis, in both animal models and patients [36–42], and GRK stoichiometry can determine whether a GRK responds [42], the effect of this differential expression of GRK2 on physiological responses that leads to T-cell behaviors, such as CCR7-induced chemotaxis is important to define.

In this study, we determined that GRK2 plays a role in CCR7 activation that leads to biased signaling in T cells during ligand-induced CCR7 internalization, preferential activation of Gα_i2_ or Gα_i3_, Ca^2+^ mobilization and chemotaxis. When CCR7 was stimulated by CCL19, GRK2 promoted kinase-dependent arrestin-3 recruitment to and internalization of CCR7; however, the extent of CCR7 internalization in the presence of CCL21 was unaffected by GRK2. Chemotaxis via CCR7 is driven by Gα_i_ proteins; therefore, we examined how Gα_I_ isoforms were regulated by GRK2. CCL19-bound CCR7 activated significantly more Gα_i3_ in the presence of GRK2, which had no effect on chemotaxis, while GRK2 led to a significant increase in activation of Gα_I2_ to CCR7 in the presence of CCL21 and promoted chemotaxis. While previous studies in our lab have shown that CCL21-induced migration requires activation of phospholipase Cγ1 (PLCγ1), GRK2 did not significantly affect the activation of this lipase. Overall, our studies provide mechanistic insight into how T cells use GRK2 to finely bias their signaling responses to CCL19 and CCL21, which function through the same chemokine receptor, CCR7.

## 2. Materials and Methods

### 2.1. Cell Lines

CEM human T-cell lymphoblastic leukemia cells, which express endogenous CCR7, were a generous gift from Dr. Iannis Aifantis (New York University, New York, NY, USA). JLAT-WT, a Jurkat e6 derivative, was a generous gift from Dr. Art Weiss (University of California at San Francisco, CA, USA) [43]. Human T-cell lines were maintained in heat-inactivated 10% fetal bovine serum (#S111150H; R & D systems, Minneapolis, MN, USA)/90% RPMI (#10013CV Corning, Corning, NY, USA)/2 mM L-glutamine (#A2916801; Invitrogen, Waltham, MA, USA). HEK293T cells (CRL-3216; Manassas, VA, USA) were maintained in heat-inactivated 10% fetal bovine serum/90% DMEM (#10102CV Corning^®^, Corning, NY, USA) 2 mM L-glutamine (cDMEM—Invitrogen, Waltham, MA, USA). All cell lines were cultured in a humidified atmosphere of 5% CO_2_/air at 37 °C.

### 2.2. Construct CRISPR and Target Sites for GRK2

GRK2 exons were scanned for sequences containing the NGG that were unique to GRK2. Forward and reverse primers flanking the target site were produced, tagged with a CACC forward-sequence (CACCGGCCCTTGGTGGAATTCTATG) and an AAAC-reverse sequence (AAACCATAGAATTCCACCAAGGGCC) adjacent to the PAM. Oligos were phosphorylated with T4 polynucleotide kinase (#M0201L; NEB; Ipswich, MA, USA), annealed by heating to 95 °C and cooling to 35 °C in 5 °C increments, and ligated to pre-digested (BsmBI (#R0580; NEB)) pLENTI-CRISPRv2 (#52961; Addgene; Watertown, MA, USA) [44] to generate pLENTI-CRISPRv2-GRK2. The double-stranded oligonucleotide served as the gRNA template to target exon 7 of human GRK2 [44].

### 2.3. Cell Transfections

HEK293T cells were seeded at 1.7 × 10^6^ cells per well in a six-well dish in cDMEM. Twenty-four hours later, the media was aspirated, cells were washed twice with pre-warmed PBS (#21–030-CM Corning^®^) and fed with 1 mL complete Iscove’s Modified Eagle’s Medium (IMEM-#10–016-CV Corning^®^)/10% fetal heat-inactivated bovine serum/90% RPMI/2 mM L-glutamine (cIMEM). Following a 3.5 h incubation, 0.5 μg of DNA was diluted into 50 μL serum-free DMEM (*sf*DMEM) (Corning^®^), as follows: (0.17 μg hCCR7-GFP, 0.17 μg GRK2 (A generous gift from Dr. Marc Caron, Duke University Medical Center, Durham, NC, USA), and 0.17 μg Arr3-RFP [45]. For the immunofluorescence studies, the following DNAs were diluted into the *sf*DMEM: 0.25 μg human CCR7 (hCCR7) (a generous gift from Dr. Robert Hromas, University of Texas Health Sciences Center San Antonio, TX, USA) fused to GFP in pEGFP-N1 and 1 μg each: pEGFP-CCR7, GRK2 (pCMV-GRK2, pGRK2-D110A, pGRK2-R587Q, or pGRK2-K220R (generous gifts from Dr. Jeffrey Benovic, Thomas Jefferson University, Philadelphia, PA, USA), 1 μg HA-arrestin-3 (A generous gift from Vsevolod Gurevich (Addgene plasmid #234806, Watertown, MA, USA)) [46]. LipoD293 (1 μL LipoD293:50 μL *sf*DMEM) dilution was added immediately to the DNA and incubated 15 min. The DNA/LipoD293 complexes were added dropwise to each well with rocking to quickly disperse the drops. The cells were incubated overnight and plated onto coverslips the next day.

### 2.4. Lentiviral CRISPR-Cas9 Knockdown of GRK2

HEK293T cells were grown to 70% confluency, co-transfected with plenti-CRISPRv2-GRK2, Δ8.9, pVsVg (Delta 8.9 and VSV-G envelope plasmids are helper lentiviral plasmids that are used for packing lentivirus) using LipoD293 (SignaGen, Frederick, MD, USA). After 24 h, fresh media was added, and the supernatant was collected 48 h later. Lentivirus was concentrated using PEG-it virus precipitation solution (#LV810A-1, System Biosciences, Palo Alto, CA, USA). Lentivirus was transduced into CEM cells or JLAT-WT-CCR7CEM cells as described by the Millipore Sigma spinoculation protocol. Transduced cells were grown for 72 h, at which time they were subjected to selection with puromycin.

### 2.5. RT-PCR

500 ng of total RNA, isolated using the RNeasy Mini Kit (Qiagen, West Caldwell, NJ, USA), was used for synthesizing first-strand cDNA using gene-specific primers (see below) and SMART^®^ MMLV Reverse Transcriptase (#639522; Clontech, San Jose, CA, USA). RNA was digested using RNAse H (#M0297; NEB, Ipswich, MA, USA). PCR was performed using EconoTaq plus (#30033–1; Lucigen, Middleton, WI, USA) with 94 °C denaturation for 3 min, followed by 35 cycles at 94 °C for 30 s, 62 °C for 30 s, and 72 °C for 1 min. GRK2 primers (Sigma Aldrich, St. Louis, MO, USA) (Forward: 5-CACCATCAACGCTGAGACAG-3, Reverse: 5-ATCTTGAGGAGCAGGCACTT-3) and as an internal control, β-actin primers (Forward: 5-GTGGCATCCACGAAACTACC-3, Reverse: 5-CCTGCTTGCTGATCCACATC-3) were used. PCR products were separated on a 3% agarose gel and visualized with ethidium bromide. Band intensities were determined using ImageJ version1.54p freeware. Relative GRK2 levels were quantified by normalizing to the β-actin internal controls.

### 2.6. Western Blots

Cells at a concentration of 10^6^ cells/time point were stimulated with 200 nM hCCL19 or hCCL21 (#361-MI-025 or #366–6C-025, respectively. R & D Systems; Minneapolis, MN, USA). Equal number of cells were lysed in RIPA (25 mM Tris-HCl (#02–004-521; JT Baker, Avantor, Radnor, PA, USA) pH 7.6, 150 mM NaCl, 1% NP-40 (#9016–45-9, Sigma Aldrich), 1% sodium deoxycholate (#30970, Sigma Aldrich) and 0.1% SDS (#75746, Sigma Aldrich), 50 mM NaF, 100 μM Na_3_VO_4_, 40 μM sodium pyrophosphate and 25 mM β-glycerophosphate (#35675, Sigma Aldrich) buffer containing HALT^™^ protease/phosphatase inhibitor cocktails (#78429; ThermoFisher), lysates were resuspended in Laemmli buffer and fractionated on a 12% SDS-PAGE, gels dried and exposed to film or transferred to PVDF overnight, and probed at 4 °C in 1 × Tris buffered saline Tween-20 (pH → 8.0) 137mM sodium chloride, 2.7mM Potassium chloride (#S29552, Sigma Aldrich), 25 mM Tris, 0.1% Tween-20), pH 7.4)/5% milk (Carnation instant milk) or 5% BSA, as per manufacturer’s instructions using a 1:1000 dilution of goat anti-CCR7 (sc-9699; Santa Cruz Biotechnology; Dallas, TX, USA), incubated with secondary HRP-conjugated antibody (#305–035-003 Jackson ImmunoResearch Labs, West Grove, PA, USA: 5000 anti-goat in 5% BSA), for 1 hr at room temperature, rinsed three times and visualized with SuperSignal^™^ West Pico PLUS Chemiluminescent Substrate (#34580; ThermoFisher; Waltham, MA, USA). For re-probing, membranes were incubated in BlotFresh^™^ Western Blot Stripping Reagent (#Sl100324; SignaGen) for 15–30 min and then rinsed extensively in 1 × TBST. Membranes were then blocked in 1X TBST for 30 min at room temperature and re-probed.

### 2.7. Receptor Internalization Assays

Assays were performed as previously described [31]. Briefly, dissociated HEK293T, CEM cells, or freshly isolated primary murine naïve T cells were rinsed in 1 x PBS, resuspended in *sf*RPMI at 5 × 10^6^ cells/mL. Cells were allowed to rest for 10 min at 37 °C, and then stimulated with 200 nM hCCL19 or murine CCL19 (R&D Systems; #440-M3–025) or hCCL21 or mCCL21 (R & D Systems; #457–6C-025). At each time point, 100 μL of cells was transferred into 1 mL ice-cold Ca^2+^/Mg^2+^ free 1X PBS (#21–040-CM, Corning^®^) to stop CCR7 internalization. Cells were maintained on ice until all time points were collected. Cells were rested for 30 min, pelleted by centrifugation at 4 °C, rinsed with ice-cold Ca^2+^/Mg^2+^ free 1X PBS, and CCR7 on the surface was labeled with either phycoerythrin (PE)-conjugated anti-murine CCR7 (4B12) (#12–1971-82; e-Bioscience; San Diego, CA, USA), PE-conjugated anti-human CCR7 (#FAB197P-100; R & D), ALEXA Fluor 700 anti-human CCR7 (#353243; BioLegend, San Diego, CA, USA) or isotype controls from the same manufacturer for 30 min on ice in Ca^2+^/Mg^2+^ free 1X PBS/2%BSA (#A3059–100G, Sigma-Aldrich, St. Louis, MO, USA). Cells with unconjugated anti-human CD197 were rinsed with ice-cold PBS and stained with FITC-anti-mouse (#F0257-.5ml; Sigma-Aldrich) for 30 min at 4 °C. Cells were rinsed twice in Ca^2+^/Mg^2+^ free 1X PBS and assayed immediately by flow cytometry on a Gallios Flow Cytometer (Beckman Coulter; Brea, CA, USA). The level of receptor remaining on the cell membrane was calculated as [mean channel fluorescence of PE-anti-CCR7 or FITC-anti-mouse at time point]/[mean channel fluorescence of PE-anti-CCR7 at time = 0 min]. Statistical significance was determined using a paired Student’s *t-*test. Data were fit using the Prism 7 software package (Graphpad Prism; Boston, MA, USA).

### 2.8. Phosphorylation Assay

CCR7 phosphorylation was assayed as described [47]. Briefly, CEM or CEM-GRK2^+/−^ were incubated with orthophosphate in phosphate-free RPMI 1640/5 mM sodium ortho-vanadate/0.2 mCi/mL [^32^P] orthophosphate, for 3 h at 37 °C in a humidified incubator, 5% CO_2_/air. Cells were treated with 200 nM hCCL19, hCCL21 or an equal volume of PBS for 10 min at 37 °C. Cells were pelleted, supernatant removed, and cells lysed by the addition of 1 mL of Brij-96 Buffer (1% Brij96 detergent (SIALP636–500G; Sigma Aldrich), 50 mM Tris-HCl (pH → 7.5), 150 mM NaCl (#S9888, Sigma Aldrich), 5mM EDTA(E4884; Sigma Aldrich), 50 mM NaF (#7681–49-4, Sigma Aldrich), 10 mM Na Pyrophosphate (#71501, Sigma Aldrich), 2 mM Na_3_VO_4_ (#567540, Sigma Aldrich), 1 mM PMSF (#329–98-6, Sigma Aldrich), (#78440; HALT^™^ Protease and Phosphatase Inhibitor Cocktail-Thermo Fisher). Lysis proceeded while rotating tubes end over end for 15 min @ 4 °C. The insoluble debris was removed by centrifugation @ 4 °C—13,000× *g* for 10 min. Pierce Protein A/G Agarose was swirled gently to resuspend and resuspended in Brij-96 lysis buffer. Using a wide-bore pipette tip, 20 μL of the resin (per IP) was transferred to each lysate and kept in suspension by gentle end-over-end mixing for 1 h. The 1.5 mL tube was pierced at the bottom with a 26G needle and placed in a collection tube. The combination was centrifuged at 4 °C @ 1000× *g* for 1 min, and the lysate flow was collected. Lysate was adjusted to 10 μg/mL anti-CCR7(MAB197; R & D)/20 μL resin, and incubated for 3 h at room temperature. The beads were centrifuged at 4 °C @ 1000× *g* for 1 min, washed three times in the Brij-96 buffer, and resuspended in elution buffer (for each IP; 50 μL of 2X Laemmlli Sample Buffer, 20 mM DTT final concentration) and incubated at 65 °C for 10 min in a dry heat bath. The 1.5 mL tube was pierced at the bottom with a 26G needle, and placed in a collection tube and eluted at 4 °C at 1000× *g* for 1 min and the eluate was collected and fractionated on a pre-run 12.5% SDS-polyacrylamide gel. Gels were dried, and ^32^P content was determined with a BIO-RAD Personal Molecular Imager (#170–9400; BIO-RAD, Hercules, CA, USA).

### 2.9. Immunofluorescence

Twenty-four hours after transfection, cells were trypsinized, rinsed, and re-plated on glass coverslips, allowed to adhere and spread for 16–24 h, then incubated with 20 nM CCL19 or CCL21 for intervals up to 30 min. Cells were fixed with 2% paraformaldehyde in phosphate buffered saline (PBS) for 15 min stained with anti-HA (#C29F4; Cell Signaling Technology, Danvers, MA, USA) at a dilution of 1:800, followed by ALEXA-647 conjugated donkey anti-rabbit (#711–605-152; Jackson ImmunoResearch Labs, West Grove, PA, USA) at a dilution of 1:500, rinsed 3x with PBS with Ca^2+^/+Mg^2+^ (Corning^®^) and mounted with ProLong Gold antifade with DAPI (#P36931; Invitrogen). Twenty later, coverslips were imaged at 63 × magnification using an oil-immersion objective on an LSM 700 laser scanning confocal microscope (Zeiss, Oberkochen, Germany). Zen 2009 6.0 software (Zeiss) was used for image acquisition, analysis and processing.

### 2.10. Nucleofections

For the fluorescence resonance energy transfer (FRET) studies [48], 2 × 10^6^ JLAT-WT cells were co-nucleofected with the following plasmids purchased from Addgene (generous gifts from Dorus Gadella). 0.25 μg G-protein Gγ2, tagged with circularly permutated Venus (cpVenus-Gγ2 (#69626) and 0.25 μg of one of three Gα_i_ subunits (Gα_i1_-mturquoise2 (#69620), Gα_i2_-mturquoise2 (#69621), or Gα_i3_-mturquoise2 (#69622)).

### 2.11. Fluorescence Resonance Energy Transfer (FRET) Measurements

Nucleofected JLATWT-CCR7 and JLAT-WT-GRK2^+/−^ cells were resuspended at 2 × 10^6^ cells/mL in Hanks Balanced Saline Solution (#14025, Gibco (ThermoFisher)). Cells were transferred to black 96-well plates at 2 × 10^5^ per well, transferred to a Fluoroskan Ascent (Thermo Scientific) and stimulated with 50nM CCL19, CCL21, or PBS. Donor emission at 490 nm and acceptor emission at 550 nm were measured every second for 2 min to generate a baseline, and for 6 min to measure ligand-induced FRET. FRET was calculated as the ratio of the ($\bar{X}\;\text{acceptor}\;\text{emission}/\bar{X}\;\text{donor}\;\text{emission}$). Each study used duplicate wells to generate measurements. ΔFRET was calculated as ${\bar{X}}_{\text{stimulated}\;\text{cells}}-{\bar{X}}_{\text{baseline}}$ in Excel and plotted in Prism. All measurements represent an average of 3 independent studies ± S.E. Significance of * *p* ≤ 0.05, ** *p* ≤ 0.01, *** *p* ≤ 0.001.

### 2.12. Mice

Mice were bred in-house or purchased from the Jackson Laboratories. The C57BL/6 GR K2^+/−^ (GRK2^+/−^) mice were generous gifts from Dr. Robert Lefkowitz (Duke University, Durham, NC, USA). The mice were genotyped as described [49,50]. PCR was used to genotype GRK2^+/−^ mice using the following primers: Primer 1: 5^′^-TGGATGTGGAATGTGTGCGAG-3^′^, Primer 2: 5^′^-GACTTCTGCCTGAACCATCTG-3^′^, and Primer 3: 5^′^-TTCGACAAATCTCCTCAATGTAT-3^′^. PCR amplification cycle was as follows: 95 °C denaturation for 3 min, followed by 32 cycles of 95 °C for 30 s, 58 °C for 45 s, 72 °C for 45 s. The reaction was terminated with a 10 min extension at 72 °C followed by a 4 °C hold. For the GRK2 PCR, DNA from wild-type mice produced a band at 800 bp, while heterozygotes produced a band at 800 (WT) and 705 bp (KO). The C57BL/6 GRK2^f/f^ (Jackson Labs #012458) [51] mice were crossed with C57BL/6-CD4-Cre mice (Jackson Labs #022071) to generate C57BL/6 GRK2^f/f^ CD4-Cre mice. All animals were maintained in the laboratory’s animal care facility under the direction of Jeana Barrow, DVM. All experiments were approved by the University of Texas at El Paso IACUC (protocol #A201310) or by the University of Kansas Medical Center IACUC (protocol 2010–1876).

### 2.13. Splenic Isolation of T Cells

Briefly, mice were euthanized with Euthaphen Euthanasia K9 100 mL C3N* (#VINV-CIII-015, Skipton, UK). Chest cavities of mice, non-responsive to toe pinch, were opened, spleens were excised, and cells were isolated by pressing the spleens through a wire screen using the black rubber end of a plunger from a 3 mL syringe. T cells were isolated by negative selection (#19851, StemCell, British Columbia, CA, USA), and viability was determined by Trypan blue exclusion. The purity of the prepared cells was confirmed by staining for naïve (CCR7+: Clone 4B12; ThermoFisher) and pan T cells (CD3ε+: Clone 17A2; Biolegend, San Diego, CA, USA), using fluorescence-activated cell sorting (FACS). Negative FACS controls included cells that were not negatively selected and an isotype control for each antibody. Cells were used immediately or allowed to proliferate overnight in the presence of RPMI/10% FBS, 40U/mL IL-2 (#402-ML-020; R&D)/IL-7 (#407-ML-005).

### 2.14. Chemotaxis Assays

Assays were performed as previously described [31]. Briefly, either JLAT-CCR7, JLAT-CCR7-GRK2^+/−^, or purified primary murine naïve T cells were re-suspended in filter-sterilized (0.2 μm^2^) assay buffer (HBSS (#14025092; ThermoFisher + 10 mM HEPES pH 7.4 (#J67485. AK; Invitrogen) with 1% BSA (#A790 Sigma Aldrich)). Cells were diluted to 4 × 10^6^ cells/mL in assay buffer and kept at room temperature during preparation of chemotaxis chambers. Polycarbonate (PC) membranes (5 μm pore size) were pre-wet with assay buffer/10 μg/mL fibronectin (Sigma #F0895). Chemokines (human or murine CCL19 and CCL21) were diluted to 200 nM in assay buffer and pipetted into the lower chamber of a chemotaxis chamber (#AP48; Neuroprobe), overlayed with PC membrane, gasket, and lid. Each condition was run in duplicate. Chemotaxis was induced for 90 min at 37 °C in a 5% CO_2_/air humidified incubator, PC membranes removed, and migrated cells counted using a hemocytometer. Migration indices (# of cells migrated to ligand/# of cells migrated without ligand) were determined with three independent studies. Significance was set as a *p*-value of * *p* ≤ 0.05, ** *p* ≤ 0.01, *** *p* ≤ 0.001.

### 2.15. Calcium Mobilization Assay

Cells were washed in HBSS and resuspended at 5 × 10^6^/mL. Four micromolar, Fluo-4 AM (#F14201, ThermoFisher)/Pluronic F-127 (#P6866, ThermoFisher) were added to cells for 45 min at 37 °C; cells were inverted at 5 min intervals during incubation. Cells were washed 3 x with HBSS and allowed to rest at RT for 45 min in the dark. Cells were resuspended in HBSS at 4 × 10^6^/mL, 100 μL plated on black 96-well plates, and after a 2 min baseline, assayed for 10 min ± 200 nM of CCL19 or CCL21 (FLUOROscan Ascent plate reader (ThermoFisher)). Fluo-4 AM Ex485 nm, Em527 nm.

## 3. Results

### 3.1. GRK2 Promotes Receptor Phosphorylation and Recruitment of Arrestin-3 to CCL19 but Not CCL21 Stimulated CCR7

**Figure 1.**
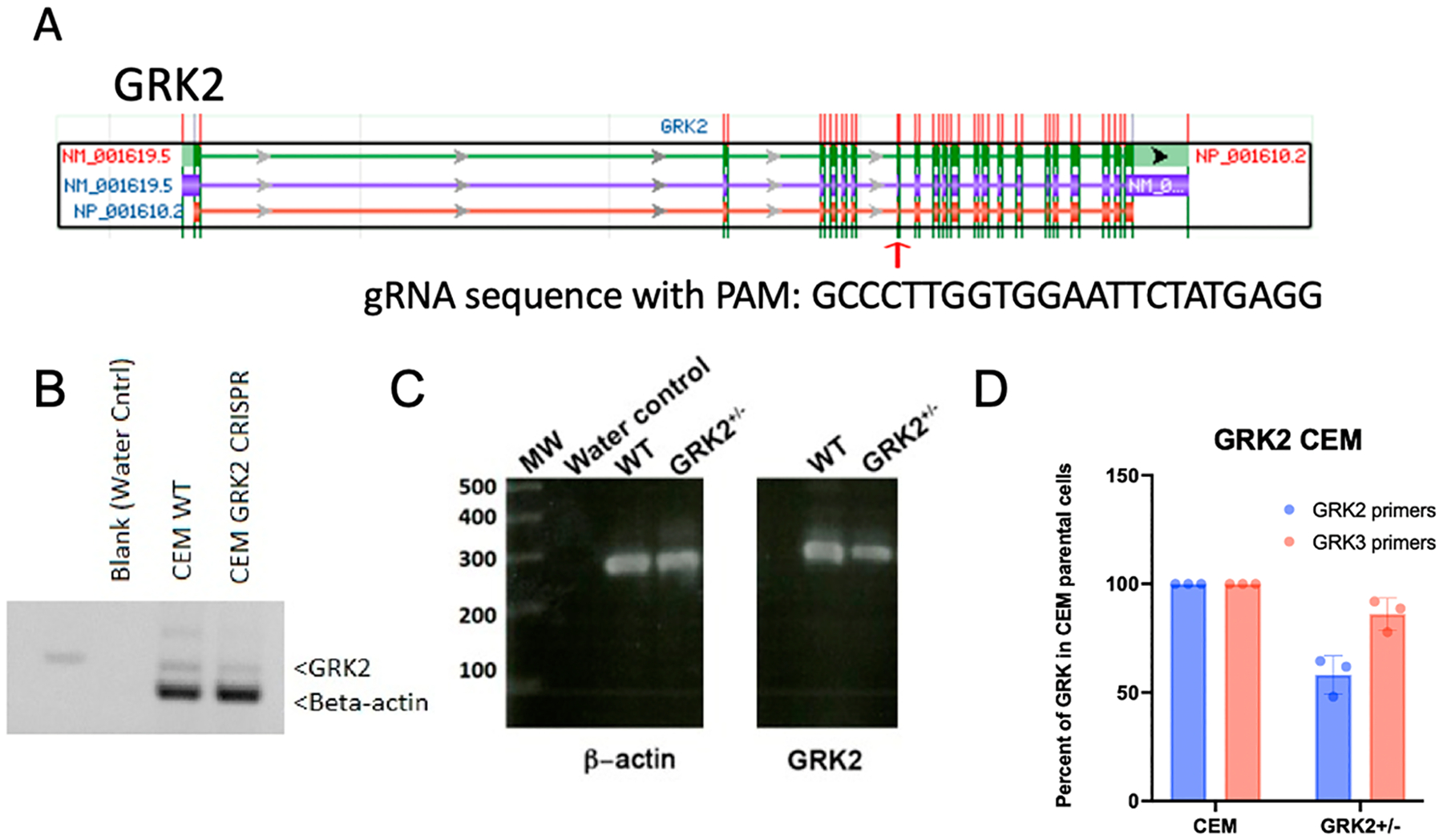
Generation of CRISPRs to disrupt GRK2 expression in CEM and JLAT-WT cells. (**A**). Genomic map of GRK2. The red arrow depicts the site of the indicated PAM sequence from which the gRNA was constructed. (**B**). RT-PCR of GRK2 in CEM, CEM-GRK2^+/−^ and (**C**). JLAT-WT, JLAT-WT-GRK2^+/−^ cells. (**D**). Quantification of GRK2 in CEM and CEM-GRK2^+/−^ cells.

Our goal is to better understand the role of GRK2 in regulating the function of CCR7 in T cells. Therefore, we used two human T-cell lines, CEM and JLATWT-CCR7, to generate two derivative cell lines in which the GRK2 locus was disrupted by transduction of pLENTI-CRISPRv2-GRK2. The GRK2 family members share 73% homology. Therefore, to ensure that pLENTI-CRISPRv2-GRK2 targeted only GRK2, we designed the gRNA to not overlap other GRKs or sites in the human genome (Figure 1A). The transduced cells were selected in the presence of puromycin and analyzed for expression of GRK2 by RT-PCR (Figure 1B–D). Using RT-PCR, we found that only heterozygote cells survived selection and were found to express approximately half of the GRK2 that the parental JLATWT-CCR7 or CEM cells did (Figure 1B–D). Only GRK2^+/−^ cells were generated (Figure 1), presumably since GRK2^−/−^ cells did not survive selection. GRK3 RNA levels remained close to WT levels (Figure 1D).

**Figure 2.**
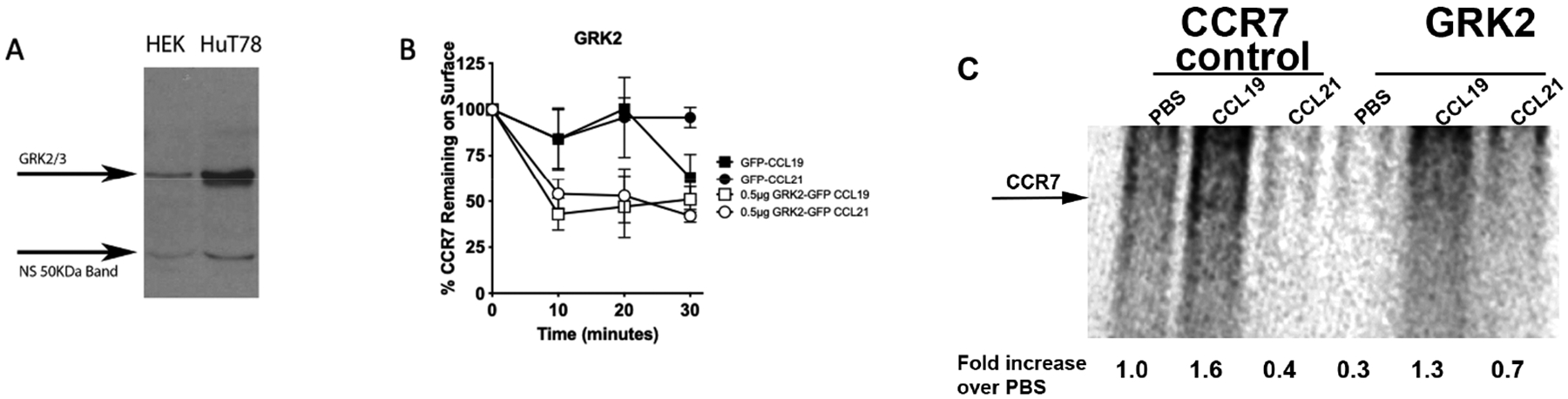
GRK2 promotes ligand-dependent internalization of CCR7 in human embryonic kidney (HEK293T) cells and CCR7 phosphorylation in human CEM T cells. (**A**) Hut 78 and HEK293T cells were lysed, fractionated by SDS-PAGE and analyzed by Western blot for expression of GRK2 using a pan GRK2/3 antibody/secondary antibody. A non-specific band (NS) served as a control for protein loading. (**B**) HEK293T cells transfected with CCR7 and GFP or CCR7 and 0.5 μg GRK2-GFP were stimulated with 200 nM CCL19 or CCL21, and CCR7 internalization was measured by FACS. Each point represents the mean of a minimum of three independent assays ± S.E. Significance was determined by comparing 2 points using a Student’s *t*-test. (**C**) Representative image of CEM or CEM-GRK2^+/−^ labeled with orthophosphate, stimulated in the presence of PBS (unst.), 200 nM CCL19 or 200 nM CCL21, fractionated by SDS-PAGE and analyzed by phosphoimager.

GRK-mediated phosphorylation of GPCRs promotes GPCR desensitization and internalization [52]. Since arrestins attach to phosphorylated intracellular loops and C-termini of activated GPCRs, we compared the contribution of GRK2 to biased arrestin binding to CCR7 and internalization of CCR7. In human T cells, ~80% of heavily phosphorylated CCR7 internalizes in the presence of CCL19 in an arrestin-3 dependent manner, while CCL21 promotes weak CCR7 phosphorylation and internalization of ~20% of CCR7, in an arrestin-3 independent manner [3,31]. Mechanisms leading to differential CCR7 phosphorylation in immune cells have been unclear [31,34,53,54], in part, because HEK293T, which do not express substantial levels of GRK2, have been used to define roles for GRK2 in ligand-dependent CCR7 internalization and signaling [30]. When compared to human T cells, which express high levels of GRK2, HEK293T cells expressed lower levels of GRK2 (Figure 2A) [30]. Therefore, to determine if the elevated levels of GRK2 expressed in T cells could promote differential internalization of CCR7 in HEK293T, we transfected HEK293T cells with human CCR7± GRK2-GFP and used flow cytometry to compare CCR7 internalization in the presence of CCL19 and CCL21 in cells ± GRK2-GFP. We found that independent of GRK2 expression, there was no significant difference between CCR7 internalization of CCL19 or CCL21 (Figure 2B). However, GRK2 promoted more internalization overall of CCR7 independent of the ligand. This suggested that although GRK2 can induce CCR7 internalization, the differential internalization observed in T cells could not be due only to the high levels of GRK2 expressed in these cells. In addition, it was unclear if GRK2 induced differential phosphorylation of CCR7 in a ligand-dependent manner.

To determine whether GRK2 promoted differential phosphorylation of CCR7, we used CEM and CEM-GRK2^+/−^ cells grown in the presence of ^32^P-orthophosphate overnight, stimulated with CCL19 or CCL21, lysed, and lysates were immunoprecipitated for CCR7. The lysates were fractionated on gels and dried so that phosphorylated receptor levels could be imaged. Stimulation of CCR7 in the parental CEM with CCL19 promoted a 60% increase in the levels of phosphorylated CCL19/CCR7 over the levels of phosphorylated CCR7 present in unstimulated cells, while CCL21 stimulation of CEM showed a 60% decrease in phosphorylation when compared to unstimulated cells (Figure 2C). In the CEM-GRK2^+/−^ cells, we observed a 70% reduction in the level of CCR7 phosphorylation in unstimulated cells, when compared to the unstimulated CEM parental cells. However, both CCL19 and CCL21 induced CCR7 phosphorylation in the CEM-GRK2^+/−^ cells (Figure 2C) when compared to unstimulated CEM-GRK2^+/−^ cells. These results demonstrate that GRK2 promotes basal levels of CCR7 phosphorylation that result in CCR7 phosphorylation in the presence of CCL19, and reduction of CCR7 phosphorylation in the presence of CCL21.

**Figure 3.**
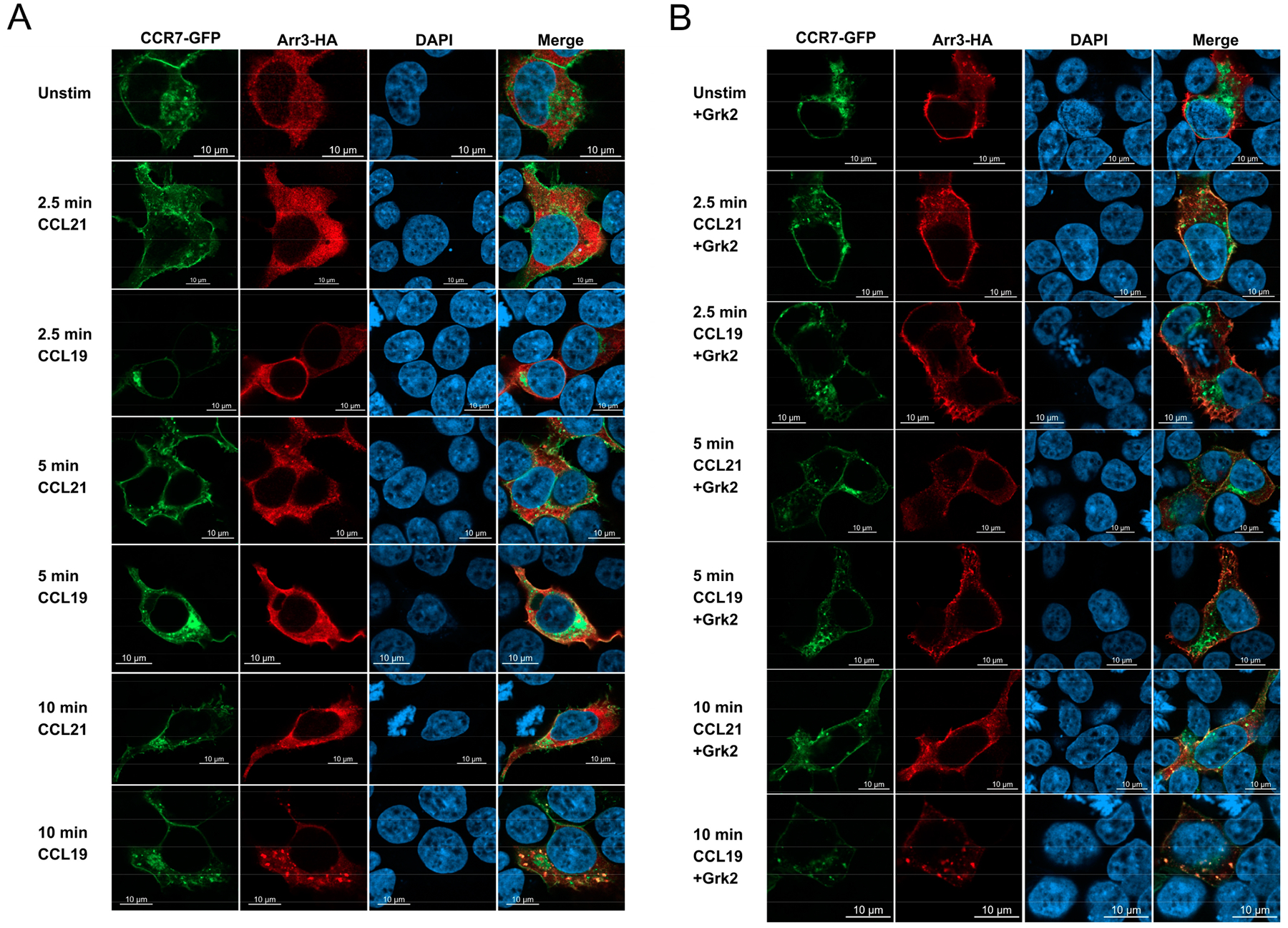
GRK2 promotes ligand-independent recruitment of arrestin 3 to CCR7. HEK293T (**A**) CCR7-GFP/Arrestin 3HA or (**B**) CCR7-GFP/Arrestin 3HA+GRK2 transfectants plated on glass coverslips were stimulated with 200 nM ligand for the indicated time intervals, fixed, permeabilized and stained for HA. Cells were imaged using a Zeiss Airyscan super-resolution microscope. Images are representative of *n* = 3 independent studies.

Since internalization of CCR7 in the presence of CCL19 is dependent on arrestin-3 [31], which binds phosphorylated GPCRs, we questioned if overexpression of GRK2 would lead to CCR7 recruitment of arrestin-3. To examine this question, we used confocal immunofluorescence microscopy of HEK293T cells transfected with CCR7-GFP (HEK-CCR7-GFP), in the presence or absence of GRK2 (Figure 3). We found that following 10 min of treatment with 20 nM CCL19, CCR7 internalized and colocalized with arrestin-3, while CCL21 did not (Figure 3A). In contrast, overexpression of GRK2 led to promiscuous CCR7 recruitment of arrestin-3 independent of the ligand (Figure 3B). We concluded that overexpression of GRK2 was sufficient to induce arrestin-3 recruitment and CCR7 internalization to both CCL19 and CCL21. The equal levels of internalization were likely due to increased phosphorylation, reflected in the recruitment of arrestin-3, which colocalized within 10 min.

### 3.2. GRK2 Kinase Activity Mediates Recruitment of Arrestin3 to CCR7

**Figure 4.**
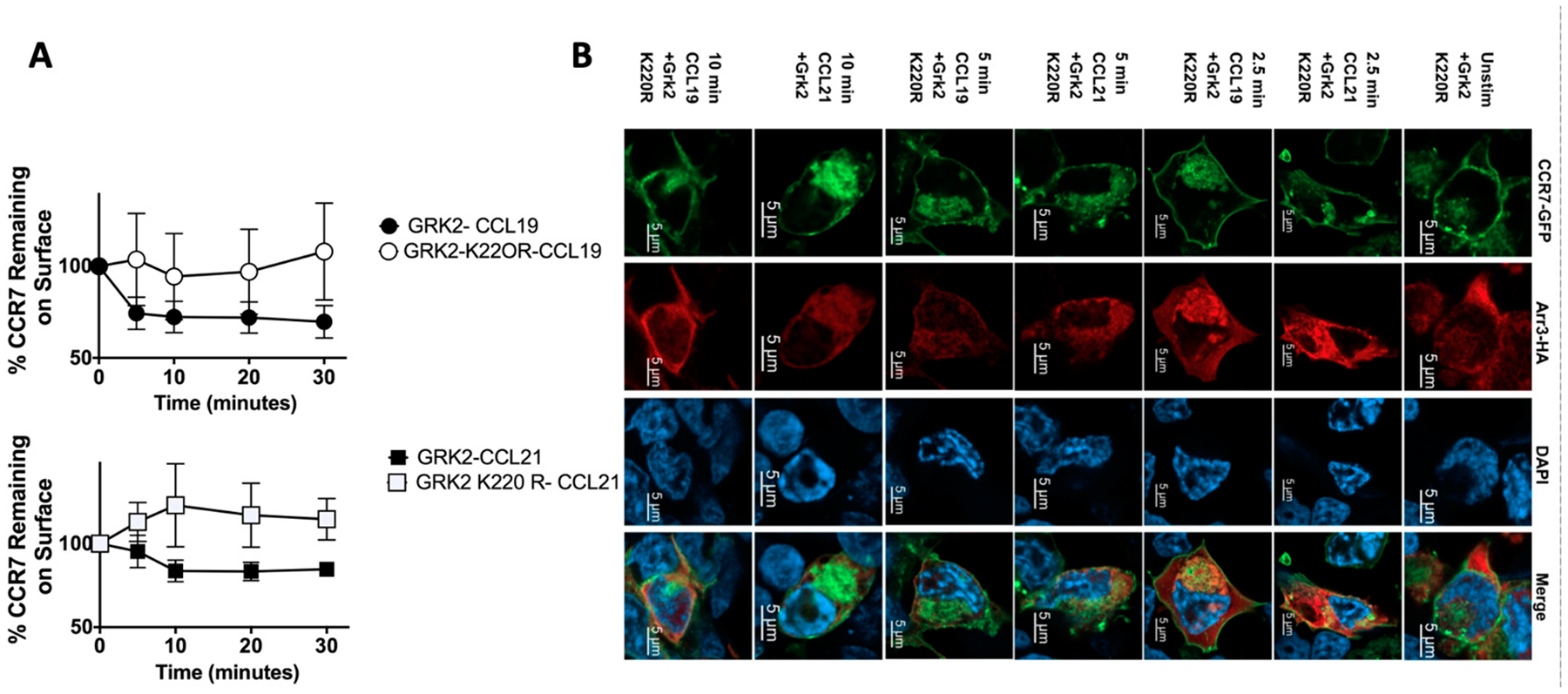
CCR7 co-localization with arrestin-3 is dependent on GRK2 kinase function. (**A**). CCR7-GFP + GRK2 or CCR7-GFP + GRK2^K220R^ +Arresin-3-HA HEK293T transfectants were allowed to internalize for 0, 10, 20 and 30 min, rinsed and stained for CCR7 and assayed by FACS. Each point is the mean ± S.E. of 3 independent assays. or (**B**) Plated on glass coverslips, stimulated, fixed, permeabilized and stained for HA. Cells were imaged using a Zeiss Airyscan confocal microscope. Images are representative of 3 independent experiments.

As GRK2 is a kinase, we questioned whether loss of the kinase function would affect receptor internalization or recruitment of arrestin 3, which promotes CCR7 internalization of CCL19, but not CCL21 [31]. In these studies, HEK293T cells were used due to their ease of transfection, and we found that loss of GRK2 kinase function by overexpression of the kinase-dead GRK2 mutant (GRK2^K220R^) prevented internalization of CCR7 in the presence of CCL19 or CCL21 (Figure 4A). Since arrestin-3 colocalizes with CCR7 during internalization induced by CCL19 (Figure 3), and loss of GRK2 resulted in loss of CCL19-induced CCR7 internalization, we wanted to determine if arrestin-3 colocalization with CCR7 was dependent on the kinase activity of GRK2 (Figure 4A). We found that in the absence of GRK2 kinase activity, CCR7 failed to internalize or to colocalize with arrestin-3 (Figure 4B).

### 3.3. GRK2 Limits Recruitment of G_αI_ Subunits to Ligand-Bound CCR7

**Figure 5.**
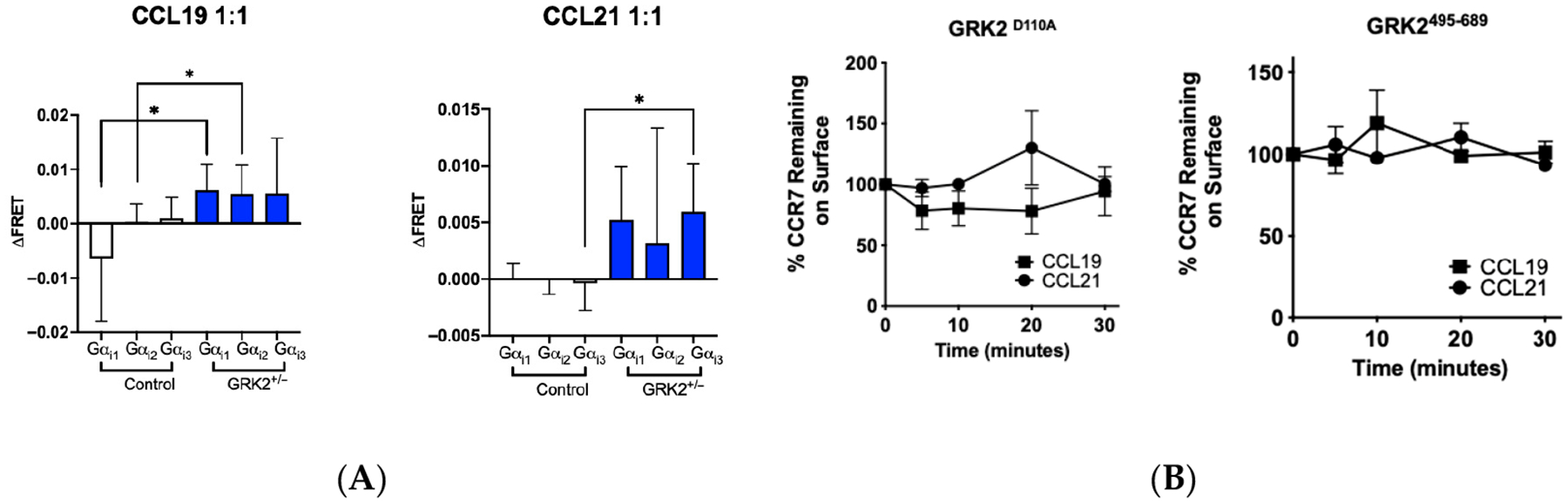
GRK2 limits G protein access to CCR7. (**A**) HEK293T cells were transfected to express Gα_i1,_ Gα_i2_ or Gα_i3_ fused to mTurquoise and cpVenus at a 1:1 ratio, stimulated with the indicated ligand, and the change in FRET was determined. (**B**). HEK293T GRK2^D110A^/CCR7 or GRK2^495−689^/CCR7 transfectants were stimulated with CCL19 or CCL21 for the indicated intervals and assayed for CCR7 internalization by FACS. Each point represents a mean ± SD of *n* = 3 or more independent studies. Statistical significance was determined by comparing two values using a Student’s *t*-test. * *p* ≤ 0.05.

Because the level of GRK kinases is higher in T cells than in HEK293T, these results call into question how the stoichiometry of GRKs in T cells affects CCR7 behavior. Therefore, we examined the effect of GRK2 on CCR7 signaling proximal to the membrane in T cells in the presence of the complement of GRKs expressed in T cells. In response to ligand binding, the GPCR initiates GTP exchange factor (GEF) activity, promoting the exchange of GDP for GTP in the Gα subunit of the associated heterotrimeric G-protein [55]. Chemokine receptors bind to Gα_i_ proteins, of which there are three different members, Gα_i1,_ Gα_i2_ and Gα_i3_; (only Gα_i2_ and Gα_i3_ are expressed in T cells) [56]. To determine if GRK2 regulated the dissociation of the heterotrimeric G-protein complex, we used the JLATWT-CCR7 and the JLATWT-CCR7-GRK2^+/−^ cells, and measured FRET of mTurquoise2-tagged Gα_i_ subunits to cpVenus-labeled Gγ_2_ (cpG_γ2_) [57]. Of the two Gα_I_ subunits expressed in T cells, CCL19 induced recruitment of Gα_I3_ subunits (Figure 5A). However, in the JLATWT-CCR7-GRK2^+/−^, FRET of Gα_i2_ subunits following stimulation with CCL19 was significantly increased, demonstrating that Gα_i2_ had reduced access to CCR7 in the presence of a full complement of GRK2 (Figure 5). In contrast, stimulation of JLATWT-CCR7 cells with CCL21 led to significantly increased FRET of Gα_i1,_ which is not expressed in T cells, and Gα_i3_, but not Gα_i2._ From these studies, we concluded that in human T cells, GRK2 promotes ligand-biased recruitment of Gα_i3_ to CCL19-bound CCR7 and Gα_i2_ to CCL21-bound CCR7.

GRK2 contains a C-terminal Gβγ binding pleckstrin homology domain which mediates membrane association, and an N-terminal regulator of G protein signaling homology (RH) domain, which interacts with Gαq subunits [58], where it suppresses signaling through Gαq [59], independent of the GRK phosphorylation activity. Half complement of GRK2 led to an increased level of Gαi associated with CCR7. Since FRET measures Gα_i-_mTurquoise2 FRET to cpVenusGγ, we speculated that GRK2 sequesters Gα or Gβγ, to limit the access of the heterotrimeric complex to the membrane. To determine if GRK2 limited CCR7 access to Gα or Gγ, we used HEK293T cells. We compared the effects of the D110A GRK2 mutant, which does not interact with Gαq, to the 495–689 C-terminal mutant, which lacks the ability to interact with Gβγ. For these studies, we used flow cytometry-based CCR7 internalization assays. We observed no difference in internalization rates of CCR7 when bound to CCL19 in contrast to CCL21, indicating that, independent of ligand, GRK2 regulation of Gαq or Gβγ access to CCR7 did not affect CCR7 internalization. We concluded that while GRK2 can sequester Gαq and Gβγ, this function did not affect internalization of CCR7 in response to binding either of its ligands.

### 3.4. GRK2 Kinase Activity Promotes the Extent of CCR7 Internalization of CCL19 and Increases the Rate of CCR7 Internalization of CCL21

**Figure 6.**
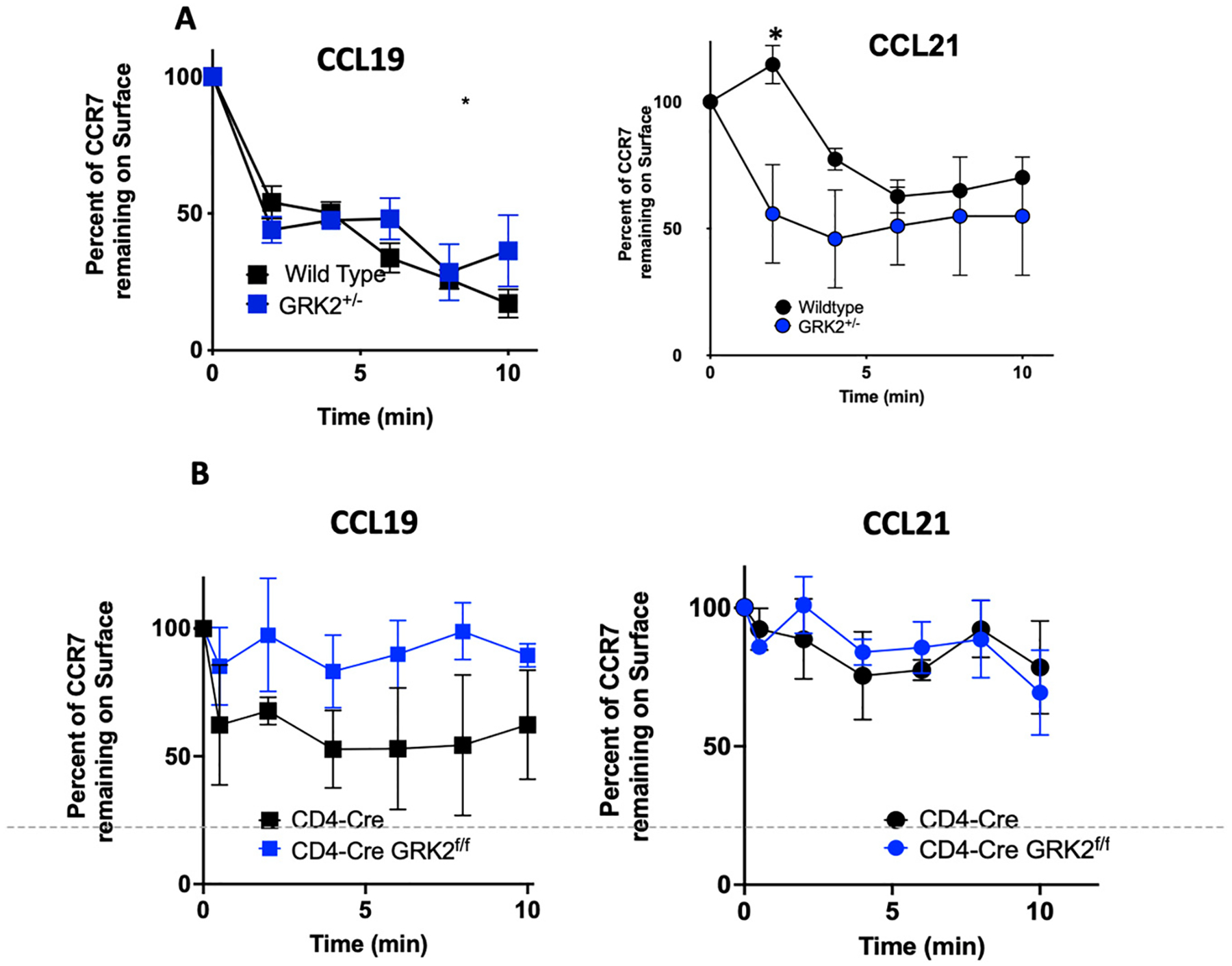
GRK2 regulates the extent of internalization of CCR7 by CCL19 and increases the rate of internalization of CCR7 by CCL21. (**A**) CEM vs. CEM-GRK2^+/−^ or (**B**) Primary CD4-Cre vs. CD4-Cre-GRK2^f/f^ T cells were induced to internalize CCR7 in the presence of 200 nM CCL19 or CCL21 for the indicated time intervals and CCR7 remaining on the surface assayed by FACS. Each timepoint is the mean ± SD of 3+ independent experiments. * *p* ≤ 0.05 was determined by a Student’s *t*-test.

Since we were unable to generate human T cell lines that were homozygously deleted for GRK2, we compared internalization of CCR7 in CD4-Cre T cells to CCR7 internalization in GRK2^f/f^-CD4-Cre T cells, in which GRK2 is homozygously deleted during T cell development (Figure 6). The CEM parental cell lines, or CD4-Cre, were used as controls. CEM cells were incubated with 200 nM CCL19 or 200 nM CCL21 and internalization was measured at 2 min intervals. In human CEM T cells, up to ~80% of CCR7 was internalized over 10 min during incubation with CCL19 [31], and this CCR7 internalization was not affected by a reduction in the levels of GRK2 (Figure 6A). In contrast, CCL21 promoted significantly more rapid internalization of CCR7 at 2 min in CEM T cells with reduced GRK2 expression (*p* < 0.05) in human CEM-GRK2^+/−^ T cells than in CEM cells (Figure 6A). In primary murine CD4-Cre T cells, similar to human CEM T cells, levels of CCR7 internalization in the presence of CCL19 were much greater than CCR7 internalization in the presence of CCL21 in the parental cell lines. However, in the absence of GRK2 in GRK2^f/f^-CD4-Cre mouse T cells, CCR7 failed to internalize in the presence of CCL19. In contrast, this complete disruption of GRK2 in the GRK2^f/f^-CD4-Cre mouse T cells (Figure 6B) had no effect on CCR7 internalization in the presence of CCL21. Therefore, we concluded that in T cells, GRK2 differentially controls receptor internalization of CCR7 following binding of CCL19 or CCL21.

### 3.5. GRK2 Promotes Migration of T Cells to CCL21 but Not CCL19

**Figure 7.**
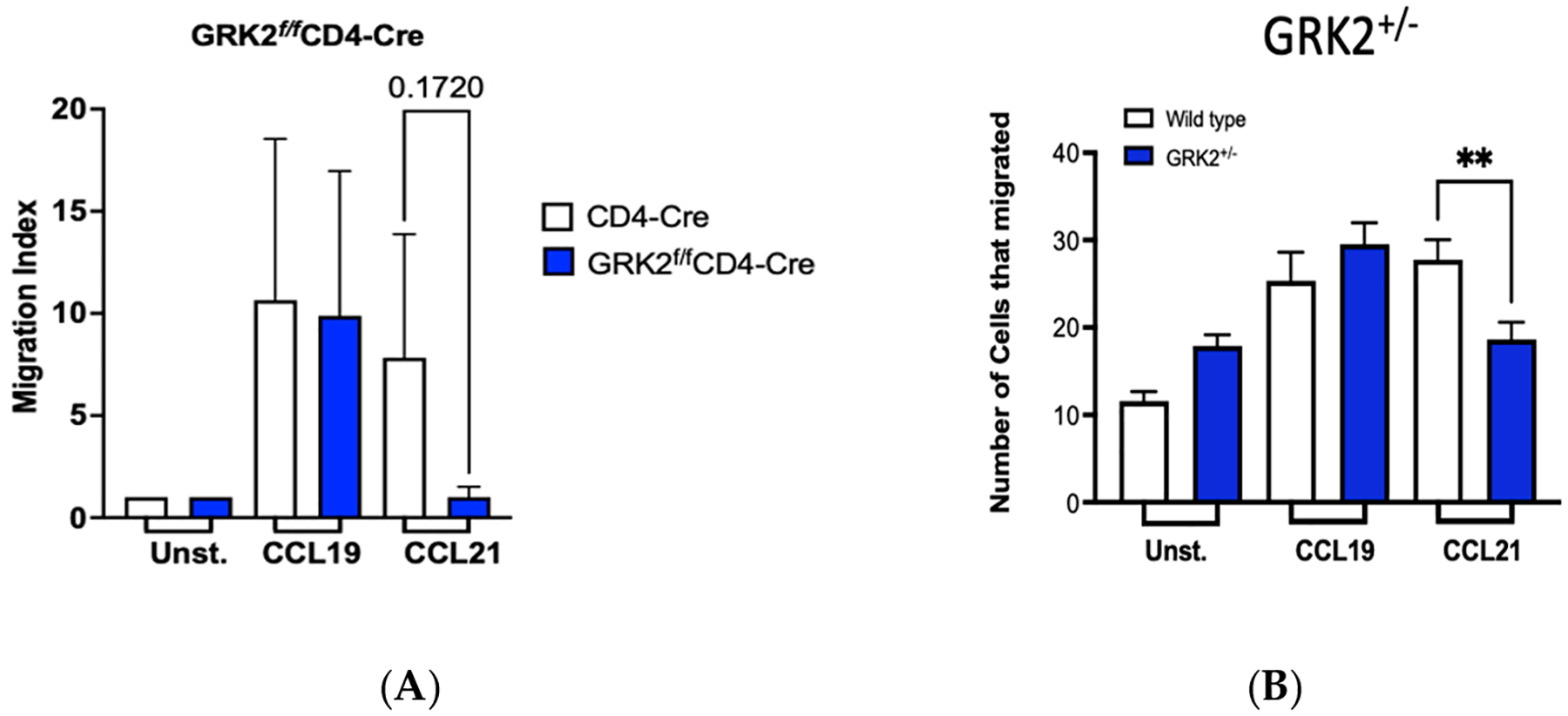
Reduction (GRK2^+/−^) or loss (GRK2^−/−^) of GRK2 attenuates T-cell chemotaxis to CCL21. Splenic T cells (GRK2^f/f^-CD4-Cre) were isolated from C57BL/6 GRK2^f/f^/CD4-Cre mice. T-cell purity was confirmed by flow cytometric analysis of CCR7 expression and the pan T-cell marker, CD3ε. Cells were used for chemotaxis immediately after isolation or after overnight incubation in culture with comparable results. (**A**) Mouse GRK2^f/f^-CD4-Cre or CD4-Cre control cells (**B**) Human JLAT-CCR7 GRK2^+/−^ T cells or GRK2^+/+^ wild type control cells were diluted to 4 × 10^6^ cells/mL, added to the top chamber and allowed to migrate across a membrane for 90 min to 200 nM CCL19 or CCL21 in the lower chamber of the chemotaxis chamber at 37 °C in a 5% CO_2_/air humidified incubator. Membranes were removed, and the number of migrated cells counted by a hemocytometer. Number of cells migrating to ligand/number of cells migrating without ligand were calculated. N ≥ 3 ± S.D. Statistical significance was determined between two conditions using Student’s *t*-test. ** *p* < 0.01.

CCR7 promotes chemotaxis of T cells to lymph nodes during homeostasis, and we and others have shown that this migration requires the activation of G*α*_i_ [18,19,54,60]. In T cells, GRK2 induces ligand-biased CCR7-signaling via G*α*_i2_ and G*α*_i3_ (Figure 5) and promotes CCR7 internalization to CCL19 but not CCL21 (Figure 6). Therefore, we questioned whether GRK2 biases CCR7 signaling that leads to migration of T cells. The roles of GRK2 in CCR7-induced migration are controversial. For instance, using primary murine T cells that lacked GRK2 expression, GRK2 was shown to have no effect on CCL19-induced migration [35], but was found to promote CCL21-induced migration [61]. For these studies, GRK2^f/f^-CD4-Cre and GRK2^+/−^ T cells were used. When the chemotaxis of primary T cells isolated from GRK2^f/f^ CD4-Cre mice to CCL19 or CCL21 was compared to that of the wild-type controls, we found that migration of GRK2^f/f^ CD4-Cre to CCL21 was reduced (Figure 7). Similarly, migration of GRK2^+/−^ T cells to CCL21 was significantly reduced when compared to T cells isolated from wild-type littermates. Overall, these studies demonstrate that GRK2 promotes migration to CCL21 but not to CCL19.

### 3.6. GRK2 Promotes Recruitment of Phospholipase C_γ_1 to the Membrane Downstream of/CLL19/CCR7

**Figure 8.**
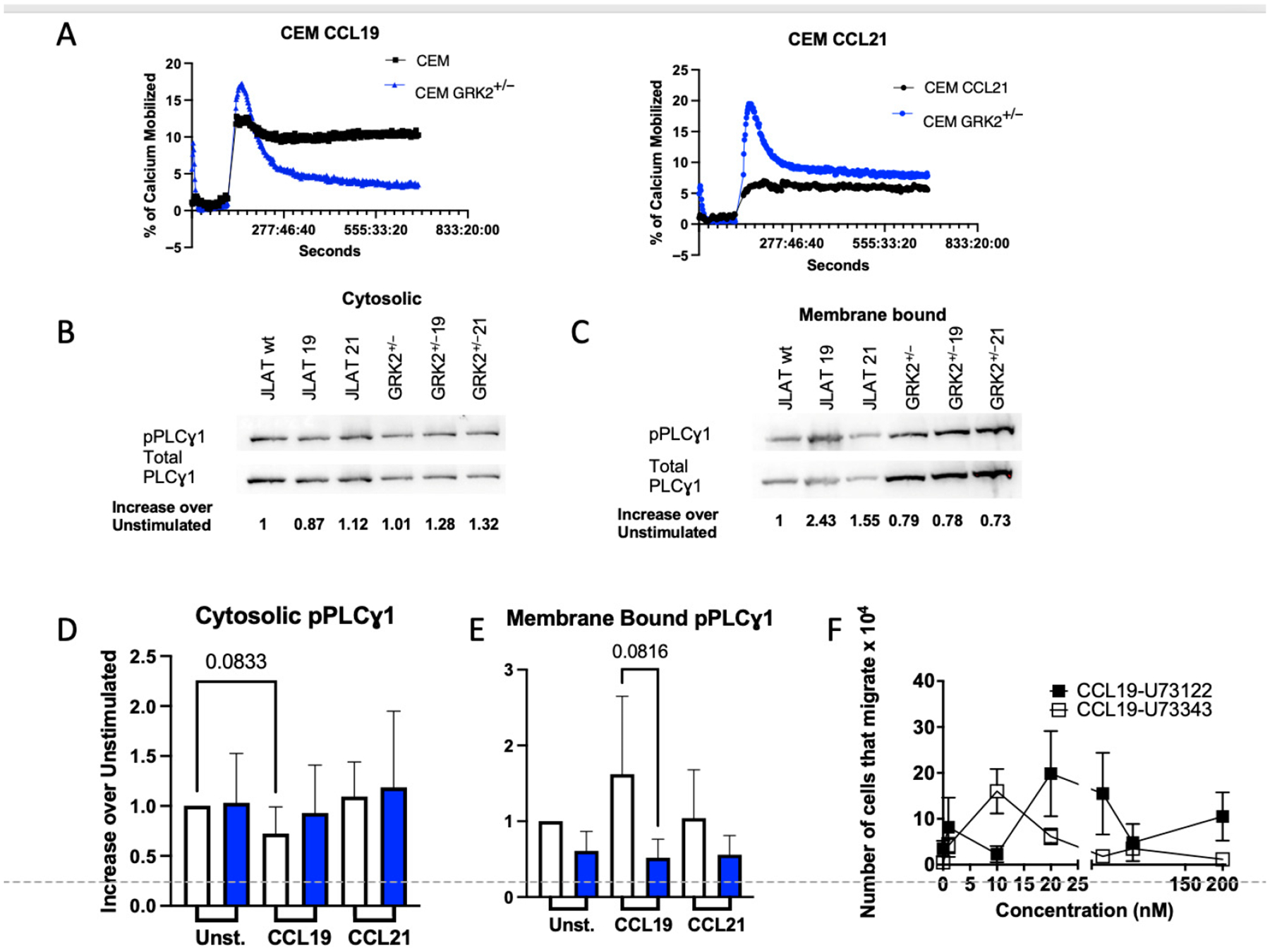
Downregulation of GRK2 in T cells results in differential ligand-induced CCR7 calcium mobilization and pPLCγ1 activation. (**A**) CEM or CEM GRK2^+/−^ T cells were pre-loaded with Fluo-4 AM at 37 °C, rinsed, and stimulated with 200 nM hCCL19 or hCCL21. Calcium mobilization was normalized to the ionomycin control maximum; data are plotted as the percentage of ionomycin control. (**B**) The 2.0 × 10^6^ JLAT-WT-CCR7 or JLAT-WT-CCR7-GRK2^+/−^ cells were unstimulated or stimulated in the presence of 200 nM hCCL19 or hCCL21 for 2 min, cells were lysed in RIPA buffer + protease/phosphatase inhibitors and (**B**) cytosolic or (**C**) membrane proteins were mixed with 2 × Laemmli buffer and fractionated on a 12% SDS-PAGE gel, transferred to PVDF and probed for pPLCγ1 according to manufacturer’s instructions. Membranes were stripped and re-probed for total PLCγ1. Western blots were repeated three times, and band intensities quantified using ImageJ freeware. pPLCγ1 band intensities were normalized to PLCγ1 and plotted as a ratio to unstimulated cells for (**D**) cytosolic or (**E**) membrane-bound proteins. JLAT-WT-CCR7 (white bars) or JLAT-WT-CCR7-GRK2^+/−^ cells (blue bars). Student’s *t*-tests were used to compare ratios between JLAT-WT-CCR7 or JLAT-WT-CCR7-GRK2^+/−^ cells for each condition. *p*-values were provided for *n* = 4 independent experiments, ±S.D. (**F**) Primary naïve murine T lymphocytes were purified, and preincubated in the presence of 2 μM U73122 or U73343 for 20 min and stimulated to migrate in the presence of increasing concentrations of CCL19 (10 nm to 2 μm) through 5-μm pore fibronectin-coated membranes (Mean of *n* = 3 ± SEM).

Signaling through CCR7 mobilizes Ca^2+^, which can activate Rac signaling upstream of cell migration [62]. We have shown that CCL21 promotes activation of phospholipase Cγ1 (PLCγ1) via phosphorylation, which mobilizes Ca^2+^ and is required for chemotaxis of T cells to CCL21 [54]. Since GRK2 promotes migration of T cells to CCL21 (Figure 7), we questioned whether signaling through PLCγ1 is regulated by GRK2 downstream of CCL21 activation of CCR7. To this end, we compared Ca^2+^ mobilization in the CEM and CEM-GRK2^+/−^ T cells. We found that GRK2 is required to maintain CCL19-induced Ca^2+^ mobilization levels. In contrast, during CCL21-induced Ca^2+^ mobilization, GRK2 expression is required for peak Ca^2+^ release, but not sustained Ca^2+^ mobilization. We used Western blots to quantify phosphorylated PLCγ1 (pPLCγ1), which was normalized to total PLCγ1 in JLAT-WT-CCR7 and JLAT-WT-CCR7-GRK2^+/−^ cells that were stimulated with CCL19 or CCL21. Levels of activated cytosolic PLCγ1 were limited by GRK2 expression independent of ligand. In contrast, levels of membrane-bound activated PLCγ1 were enhanced by GRK2, with levels of membrane-bound PLCγ1 approaching significance downstream of CCL19 (Figure 8). To confirm that CCL19-mediated migration was not regulated by PLCγ1, we used the pan PLC inhibitor U73122 (Figure 8F) and found that migration via CCL19 is not dependent upon activation of PLC.

## 4. Discussion

GRK2 has important roles in phosphorylating agonist-activated GPCRs, typically resulting in the recruitment of a β-arrestin, receptor internalization, and desensitization [7,63–65]. GRK2 can mediate phosphorylation and internalization of several GPCRs, including the β_2_-adrenergic receptor, the M_1_, M_2_, and M_3_ muscarinic acetylcholine receptors and α_2_-adrenergic receptors; indeed, GRK2 is a key regulator of cardiac muscle contraction and heart rate [66–74]. These receptors play pivotal roles during inflammatory responses [75–79]. In this study, along with other published studies, we show that T cells express high levels of GRK2, which phosphorylates CCR7 in response to stimulation with CCL19 but not CCL21, which controls downstream signaling [80–84].

CCR7 is expressed in T cells, activated B cells, monocytes, and neutrophils [18–20], where it promotes cell migration to the secondary lymphoid organs [85]. Our goal was to determine the role of GRK2 in ligand-induced CCR7 internalization and signaling leading to migration of T cells. We found that, similar to what has been reported for GRK2^f/f^ CD4-Cre T cells [61], both GRK2^f/f^ CD4-Cre, which lack GRK2 expression, and GRK2^+/−^ murine T cells migrated at reduced levels when compared to WT CD4-Cre T cells (Figure 7). Since Gα_i_ promotes chemotaxis via chemokine receptors, we examined the role of GRK2 in regulating Gα_i_ activation (Figure 4). We found that while GRK2 regulates activation of Gα_i1_ and Gα_i2_ subunits downstream of CCL19, GRK2 primarily activated Gα_i3_ downstream when CCL21 was bound to CCR7. We speculate that since we see significantly increased recruitment of Gα_i3_ to CCR7 in the absence of GRK2, compared to GRK2-replete cells, GRK2 limits CCR7 activation of Gα_i3,_ or at the very least balances it with activation of Gα_i2._ This balance may be required for sustained migration of cells to CCL21, which internalizes only ~10% of the CCR7 from the surface of the cell (Figures 1 and 5).

In human T cells grown in vitro, we replicated our primary T cell results and showed that a ~50% reduction in GRK2 expression had no effect on CCL19 and CCL21-induced CCR7 internalization. Consistent with previous reports, ligand-induced CCR7 internalization occurred to a greater extent in human T cells compared to primary mouse T cells [34]. The lack of effect of GRK2 on CCR7/CC19 internalization in human T cells could be species-specific. Although GPCR internalization generally requires receptor phosphorylation, GPCRs often have a low basal level of phosphorylation. Indeed, protein kinase C maintains basal levels of CCR7 phosphorylation in T cells [3]. Thus, it is possible that other kinases present in T cells may work with GRKs to regulate receptor internalization. However, the overall requirement for phosphorylation of the C-terminus of CCR7 in regulating CCR7 internalization in B cells is unclear since B cells that expressed exogenous CCR7, which lacked a C-terminus, internalized CCR7 almost as efficiently as full-length CCR7 [86]. We observed a low basal level of CCR7 phosphorylation with little change in phosphorylation levels of CCR7 10 min after stimulation with CCL21, and higher levels of ligand-CCR7 phosphorylation in cells stimulated with CCL19, corresponding to the greater extent of CCR7 endocytosis. However, GRK2^+/−^ T cells had a similar level of ligand-induced internalization of CCR7. These results may reflect that other GRKs are important for phosphorylation to regulate the trafficking of CCR7 within endosomes.

Our study focuses on the effects of GRK2 signaling in T cells in response to CCR7 binding to full-length CCL19 and CCL21. Structurally, CCL19 lacks the highly basic, 37 amino acid positively charged C-terminus that is present in CCL21 [87], which plays an autoinhibitory role in the ability of CCL21 to activate CCR7 and also tether CCL21 to glycosaminoglycans (GAG) at the surface of cells [88]. The auto-inhibited structure of CCL21 can be released in the presence of free polysialic acid or potentially via interaction with polysialylated N- and O-glycans present on CCR7 on the surface of activated dendritic cells, where the sialylation releases the locked state of CCL21 [88,89]. Interestingly, a third, naturally occurring CCR7 ligand is generated in the presence of plasmin [90] or proteases released by activated dendritic cells during an immune response [91], termed CCL21^Tailless^ [29]. This form of CCR21 lacks the auto-inhibitory C-terminus and is therefore released from the locked state. CCL21^Tailless^, is a more potent form of CCL21 [88]. Without the 37-residue C-terminus, CCL21^Tailless^ is no longer immobilized by GAG and diffuses more readily, and may therefore, be more readily available to immune cells expressing CCR7. Following binding of CCL21^Tailess^, CCL21 or CCL19 ligand-activated CCR7 is internalized from the surface, where continued signaling depends on the allosteric interactions between CCR7 and the N-terminus of each chemokine [92]. Notably, unlike CCL21, CCL21^Tailess^ promotes recruitment of arrestin-3 to CCR7, implicating a GRK family member in phosphorylating the CCR7 C-terminus. More importantly, determining the signaling mechanisms that are regulated by CCR7-bound to CCL21, CCL19, or CCL21^Tailless^ could provide important clues to molecular mechanisms that promote biased signaling within vesicles of internalized CCR7. In addition, it is reasonable to speculate that the auto-inhibited structure of CCL21 also limits CCR7 activation in T cells, resulting in the diminished signaling that we have observed in this study and in our previous work [34,53]. In future studies, it will be important to determine if CCR7 signaling induced by CCL21^Tailless^ is also regulated by GRK2 in T cells, and if so, how it overlaps with the signaling pathways that we have identified in this study.

In an experimental autoimmune encephalomyelitis (EAE) murine model of multiple sclerosis (MS), the premature onset of EAE in GRK2^+/−^ mice was associated with increased CNS infiltration by T cells; GRK2^+/−^ mice, however, did not relapse and overall had an attenuated inflammatory response compared to WT mice [36]. We observed an increase in the baseline migration of unstimulated GRK2^+/−^ T cells, which could account for the increased initial invasion of the CNS. The absence of relapse could be the result of the lack of chemotactic activity of these GRK2^+/−^ T cells into the brain during disease progression [36,93], and could indicate that GRK2 downregulation is important for MS commencement and progression.

Although the importance of GRK3/GRK6 in ligand/CCR7 signaling has been demonstrated previously [30,42], we have shown that GRK2 is an important regulator of ligand-biased CCR7 signaling as well. Moreover, the stoichiometry of GRK2 to other GRKs in the cell can regulate signaling of CCR7 at the cell membrane or have downstream functional consequences that affect migration of T cells. Since GRK2 has the shortest half-life of the GRKs and is rapidly degraded by proteasomes [38], fluctuations in GRK2 levels in vivo are likely to affect CCR7 chemotaxis of T cells to lymph nodes, an important role for GRK2 in T cells that is often overlooked. Considering the ubiquitous distribution of GRK2 and the large number of GPCRs and non-GPCRs, such as insulin-like growth factor 1 receptor and epidermal growth factor receptor [94] that are dependent on GRK2 kinase activity, it is probable that many of the physiological manifestations of GRK2 are stoichiometrically dependent.

## Data Availability

The data presented in this study are available on request from the corresponding author.

## Abbreviations

BSA: Bovine Serum Albumin
CCR7: C-C Chemokine Receptor 7
CCL19: C-C Chemokine Ligand 19
CCL21: C-C Chemokine Ligand 21
CNS: Central Nervous System
CpVenus: Circularly permutated Venus fluorescent protein
CRISPR: Clustered Regularly Interspaced Short Palindromic Repeats
DMEM: Dulbecco’s Modified Eagle’s Medium
EAE: Experimental Autoimmune Encephalomyelitis
FACS: Fluorescence Activated Cell Sorting
FRET: Fluorescence Resonance Energy Transfer
GPCR: G Protein-Coupled Receptor
GRK: G protein-coupled Receptor Kinase
gRNA: Guide RNA
HBSS: Hanks Balanced Saline Solution
HEK293T: Human Embryonic Kidney cells 293 large T antigen
IACUC: Institutional Animal Care and Use Committee
JLAT-WT: Jurkat e6 LAT CRISPR Deletion; reconstituted with wildtype LAT
IMEM: Iscove’s Modified Dulbecco’s Medium
MMLV: Moloney Murine Leukemia Virus
MS: Multiple Sclerosis
PAM: Protospacer Adjacent Motif
PLCγ1: Phospholipase Cγ1
RPMI: Roswell Park Memorial Institute (Media)
TBST: Tris Buffered Saline Tween
WT: Wild Type
